# Integrating a dynamic central metabolism model of cancer cells with a hybrid 3D multiscale model for vascular hepatocellular carcinoma growth

**DOI:** 10.1038/s41598-022-15767-6

**Published:** 2022-07-20

**Authors:** Alexey Lapin, Holger Perfahl, Harsh Vardhan Jain, Matthias Reuss

**Affiliations:** 1 Stuttgart Research Center Systems Biology, University Stuttgart, Stuttgart, Germany; 2 Department of Mathematics and Statistics, University of Minnesota Duluth, Duluth, MN USA; 3 Institute of Chemical Process Engineering, University Stuttgart, Stuttgart, Germany

**Keywords:** Computational biology and bioinformatics, Systems biology, Oncology, Mathematics and computing

## Abstract

We develop here a novel modelling approach with the aim of closing the conceptual gap between tumour-level metabolic processes and the metabolic processes occurring in individual cancer cells. In particular, the metabolism in hepatocellular carcinoma derived cell lines (HEPG2 cells) has been well characterized but implementations of multiscale models integrating this known metabolism have not been previously reported. We therefore extend a previously published multiscale model of vascular tumour growth, and integrate it with an experimentally verified network of central metabolism in HEPG2 cells. This resultant combined model links spatially heterogeneous vascular tumour growth with known metabolic networks within tumour cells and accounts for blood flow, angiogenesis, vascular remodelling and nutrient/growth factor transport within a growing tumour, as well as the movement of, and interactions between normal and cancer cells. Model simulations report for the first time, predictions of spatially resolved time courses of core metabolites in HEPG2 cells. These simulations can be performed at a sufficient scale to incorporate clinically relevant features of different tumour systems using reasonable computational resources. Our results predict larger than expected temporal and spatial heterogeneity in the intracellular concentrations of glucose, oxygen, lactate pyruvate, f16bp and Acetyl-CoA. The integrated multiscale model developed here provides an ideal quantitative framework in which to study the relationship between dosage, timing, and scheduling of anti-neoplastic agents and the physiological effects of tumour metabolism at the cellular level. Such models, therefore, have the potential to inform treatment decisions when drug response is dependent on the metabolic state of individual cancer cells.

## Introduction

Cellular metabolism has long been recognized to play an important role in cancer progression and response to treatment^1–3^; however, it has only been promoted to an *emerging hallmark* in the most recent revision of the Hallmarks of Cancer^4^. The attribute “emerging” reflects some ambiguity, being neither core, nor enabling. Nonetheless, the unique metabolic features of cancer have been a driving force for many important and thought-provoking research in cancer therapeutics in recent years^5–9^. Unfortunately, translating much of this knowledge into a major therapeutic breakthrough remains a critical challenge. Mathematical modelling is now recognized as a valuable tool with which to elucidate the various mechanisms that underlie a growing tumour’s response to treatment^10^, and is therefore in a unique position to identify novel therapeutic targets that exploit our knowledge of cancer metabolism. Indeed, there exists a vast body of literature on mathematical and systems biology models of cancer growth and response to treatment (for recent reviews, see^11–24^).

In particular, a commonly used framework for mathematical modelling of cancer cell metabolism is based on flux balance analysis^25–31^. In this top-down approach, the results of “omic” investigations (genome, transcriptome, proteome, metabolome and sometimes flux measurements) inform flux-balanced metabolic pathways. Although these models have contributed greatly to our understanding of metabolic pathways within cancer cells, they suffer from some limitations. For instance, this approach does not adequately capture the effects of cellular heterogeneity within a tumour, or of interactions between cells and the tumour microenvironment. Furthermore, these models are static and fail to capture key system dynamics such as temporal and spatial heterogeneities that arise due to environmental fluctuations. Finally, flux balance analysis (FBA) requires not only the stoichiometry of the network, but also an appropriate objective function and, possibly, further constraints. For instance, in the growth of microorganisms, the most consistent optimal criteria are maximising biomass yield per flux unit or maximising ATP yield per flux unit. However, in the case of cancer growth, the objective functions are typically more complex, including multi-objective optimization problems (optimization involving more than one objective function). We remark that some of these constraints may be overcome by application of ^13^C metabolic flux analysis^32–36^.

Equally, bottom-up approaches based on the kinetics of individual reactions have been used to generate testable predictions at the macro-scale from dynamic models of pathways and networks^37–48^ in microorganisms and cell cultures. Experimental methods essential for identifying the in vivo kinetics that inform these models, follow a stimulus–response methodology wherein cells grown in culture are disturbed by fast changes in extracellular glucose concentration. The resultant dynamic responses of intra- and extracellular metabolites are then measured. Two different approaches have been used to infer dynamic models from this data. In the modular approach, the metabolic network is decomposed into manageable subunits, and experimental measurements determine the functional forms and parameters of the kinetic equations^37,38^. The second approach utilizes optimal control methods for model simplification, such as lin-log approximations^49–51^. Examples of the application of lin-log kinetics for the simultaneous estimation of model parameters include the whole cell metabolic network dynamics of *E. coli*^52^ and a dynamic model for the central metabolism of HEPG2 liver cancer cells^53^.

Our objective here is to better understand how known metabolic processes occurring in individual cancer cells inform tumour-level metabolic dynamics. Specifically, we propose integrating experimentally validated dynamic models of the central metabolism of HEPG2 cancer cells within spatially resolved multiscale models of hepatocellular cancer growth. This will allow us to predict the spatio-temporal dynamics of key metabolites within a growing vascular tumour. Such models can, in the future, inform treatment decisions when drug response is dependent on the metabolic state of individual cancer cells. Indeed, specialized articles on cancer cell metabolism often acknowledge the need for such an integrated approach^54,55^, but attempts to develop the necessary quantitative framework have not been previously reported.

We begin by extending previously published hybrid, multiscale models of vascular tumour growth (see Owen et al.^56^ and Perfahl et al.^57^). These model couple blood vessel formation or angiogenesis in response to a growing tumour’s nutritional demands with blood flow, nutrient transport, and the nutrient-dependent processes of cellular proliferation, quiescence and apoptosis. We supplement this 3D framework with external glucose balance, including the transport of glucose from the blood vessels, its diffusion through the interstitium, and its uptake by cancer and healthy cells. The metabolism of internalized glucose is explicitly included by integrating within this framework, a dynamical model of the central metabolism in HEPG2 cells, developed by Maier et al.^53^. This model has been experimentally validated by quantitative measurements of metabolite concentrations under dynamic conditions (stimulus–response experiments) and metabolic flux distributions obtained from transient ^13^C flux analysis^32,33^. Additionally, we propose a new functional form for the probability of successful anastomoses during angiogenesis in 3D, which is based on actual experimental observations^58–61^. Specifically, we allow this probability to be a function of distance between sprout tips, rather than simulating anastomosis in a more phenomenological fashion where it occurs when two sprout tips (or a sprout tip and an existing vessel) meet simply due to motion on the grids of the cellular automaton model.

Simulations of the integrated multiscale model predict for the first-time, 3-dimensional concentration profiles of metabolites within a growing vascular tumour. Longitudinal sampling from the simulated time-series of tumour development allows us to create images of the spatial–temporal distributions of these metabolites. These results illustrate the response of tumour and normal cells to various glucose and oxygen uptakes rates, including the extreme situations of normoxic, hypoxic and anaerobic conditions.

The remainder of this paper is organized as follows. In the “Methods” section, we present our mathematical model and the underlying computational framework. In “Results” we present simulation results and conclude with a discussion on the significance of our findings in “Conclusions”.

## Methods

### 3D multiscale hybrid model of vascular tumour growth

**Figure 1:**
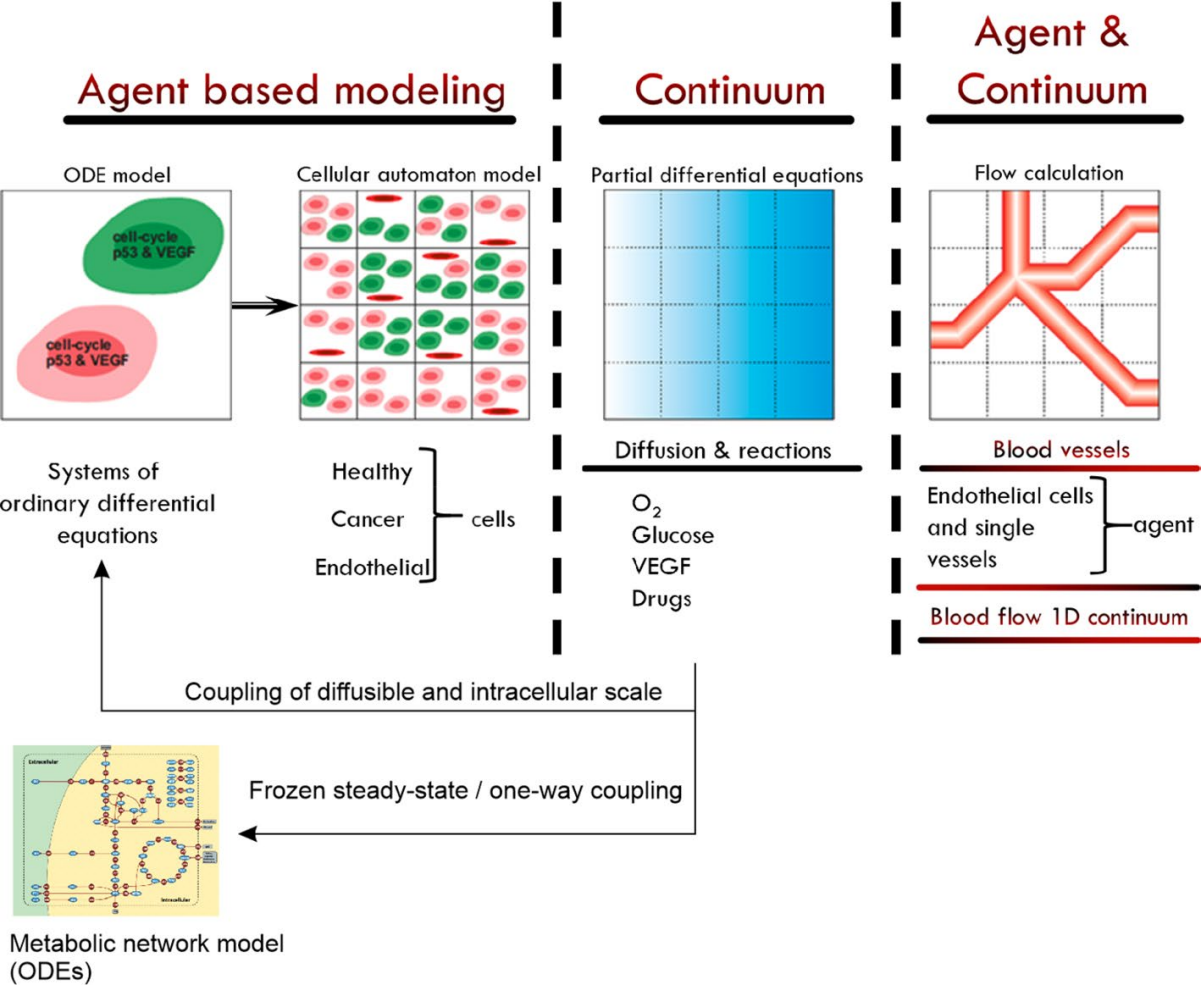
Multiscale model overview^57^. Metabolic network model reprinted from^53^ under a CC BY license, with permission from BMC Systems Biology, original copyright 2010.

The 3-dimensional model of vascular hepatocellular carcinoma growth is based on the multiscale hybrid models of tumour growth proposed by Owen et al.^56^ and Perfahl et al.^57^. The model integrates four distinct scales: sub-cellular, cellular, diffusible species, and a vascular layer, as shown in Fig. 1. Model species interact with each other according to predefined rules and coupling mechanisms as described in^56,57^. Here, we briefly summarize how this hybrid framework works. We refer the reader to^56,57^ for further details.

The sub-cellular scale is deterministic and considers three different cell types (normal cells, cancer cells and endothelial cells). Intracellular behaviour is described by ordinary differential equations (ODEs) for intracellular VEGF production, p53-, CDH-, p27-activity, and progression through the cell cycle. These, in turn, govern rule-based cellular behaviour such as division, quiescence and apoptosis. In our formulation, each cell is a stochastic agent whose behaviour is simulated via a cellular automaton model, which describes cell–cell interactions and cell movement. The extracellular concentrations of diffusible species—VEGF and oxygen—are determined by partial differential equations (PDEs) of the reaction–diffusion type, solved to quasi steady-state. In^62^ this modelling-layer has been further extended for the description of distributions of drugs. The vascular network comprises vessel segments connecting adjacent nodes on the simulation lattice, with defined inflow and outflow nodes, and prescribed pressures. The vascular network evolves as follows. Vessel sprouts form with a probability that increases with local VEGF concentration. Each sprout is an individual agent in our model whose movements are described by a biased random walk. As sprout tips migrate up concentration gradients of VEGF, they lay down behind them microvessels contiguous with the parent vessel. These new vessels become functional when a circulation loop is completed. This is realised when the guiding sprout tip anastomoses with other sprouts tips or vessels.

Challenges in extending the 2D framework to three dimensions were examined by Perfahl et al.^57^. A crucial observation was that successful anastomoses during angiogenesis are less probable in 3D, since the moving sprout tips have an additional degree of freedom. Without anastomoses, circulation loops in new vasculature cannot be completed, and blood supply cannot be established. One limitation of the 2D/3D model is that the frequency of anastomoses is a function of grid size. This simplistic assumption permitted enough successful anastomosis encounters in two dimensions. However, the corresponding probability of successful encounters in three dimensions is reduced, leading to less efficient angiogenesis and inhibited tumour growth. We remark that reducing the grid size in three dimensions, or assuming a higher initial vessel density did not entirely ameliorate this limitation.

In contrast to the aforementioned approach, we instead propose the following formulation wherein the probability of anastomosis is taken to be a function of the distance $\Delta x$ between two sprout tips or a sprout tip and an existing functional vessel.

1 $$P_{anastomosis} = 1 - \Delta x/\Delta x_{\max } ,\quad {\text{for}}\;\Delta x < \Delta x_{\max } ;\quad0,\ {\text{otherwise}}$$

where $\Delta {x}_{max} = 100 \mu \mathrm{m}$ is a constant representing the maximum distance of possible anastomosis. We remark that the above equation is underpinned by biological observations. Though a pivotal step in angiogenesis, the mechanisms driving vessel anastomosis are poorly understood. Much research has focused on elucidating how a sprout is guided in the direction of other vessels. There is emerging evidence that when sprout tips are separated by short distances, signal transduction in sprout tip cells guides them towards each other, increasing the probability of anastomosis^58–60^. Further, Moreira-Soares et al.^61^ demonstrated how vessel sprouts are guided towards each other even at larger scales of distance. These authors argued that such mechanisms are particularly relevant in three-dimensional space where “without it the network has a reduced number of anastomosis”. Practically, a successful anastomosis event is implemented as follows. A random number $\xi$ is generated uniformly from [0, 1], and if $\xi \le {P}_{anastomosis}$, anastomosis occurs, while if $\xi >{P}_{anastomosis}$, anastomosis does not occur. Consequently, the new random number approach for anastomosis leads to a greater velocity of migration of the vessel cells and increases the probability of anastomosis compared to the original model^57^. The algorithm for the random number generator is based on^63^.

In order to integrate the dynamic metabolic model of HEPG2 cells within this framework, we need to explicitly include glucose as the primary metabolite. Extracellular glucose is taken as a diffusible species, that is supplied by the vasculature and taken up by tumour and healthy cells. The PDE governing the spatial distribution of extracellular glucose concentration (${c}_{Glu}$) at quasi steady-state is taken to be:

2 $${D}_{Glu}\Delta {c}_{Glu}+2\pi R(t,{\varvec{x}}){P}_{Glu}\left({c}_{Glu}^{blood}-{c}_{Glu}\right)-{r}_{uptake}I(t,{\varvec{x}})=0,$$

where: ${D}_{Glu}$ is the diffusion coefficient for glucose in the interstitium (2.0 × 10^–6^ cm^2^/s, see^64^); ${P}_{Glu}$ is the permeation coefficient for transport of glucose from the blood vessel into the interstitium; ${c}_{Glu}^{blood}$ is the concentration of glucose in blood; and $I(t,{\varvec{x}})$ is an indicator function that returns the number of cells on the corresponding lattice-site with position vector ${\varvec{x}}$, and $2\pi R(t,{\varvec{x}})$ is an indicator-function that returns the vessel radius if a vessel is present at position ${\varvec{x}}$, otherwise it returns zero. The rate of glucose uptake (${r}_{uptake}$) is represented by the following Michaelis–Menten kinetic function:

3 $${r}_{uptake}={r}_{max}\frac{{C}_{Glu}}{{K}_{M}+{C}_{Glu}},$$

where ${r}_{max}$ is the maximum rate of glucose uptake r_max_ = 3.3 (mmol/l h for cancer cells), this is an estimate from measurements in mmol/(10^6^ cells min) with hepatic cells carried out with standard six-well tissue culture plates^32^. For normal cells r_max_ = 0.66 (mmol/l h). For cancer cells as well as normal cells K_M_ = 0.2 mmol/l^65^.

The model of intracellular glucose metabolism is described below.

### Dynamic model for the central carbon metabolism in liver cancer cells

**Figure 2:**
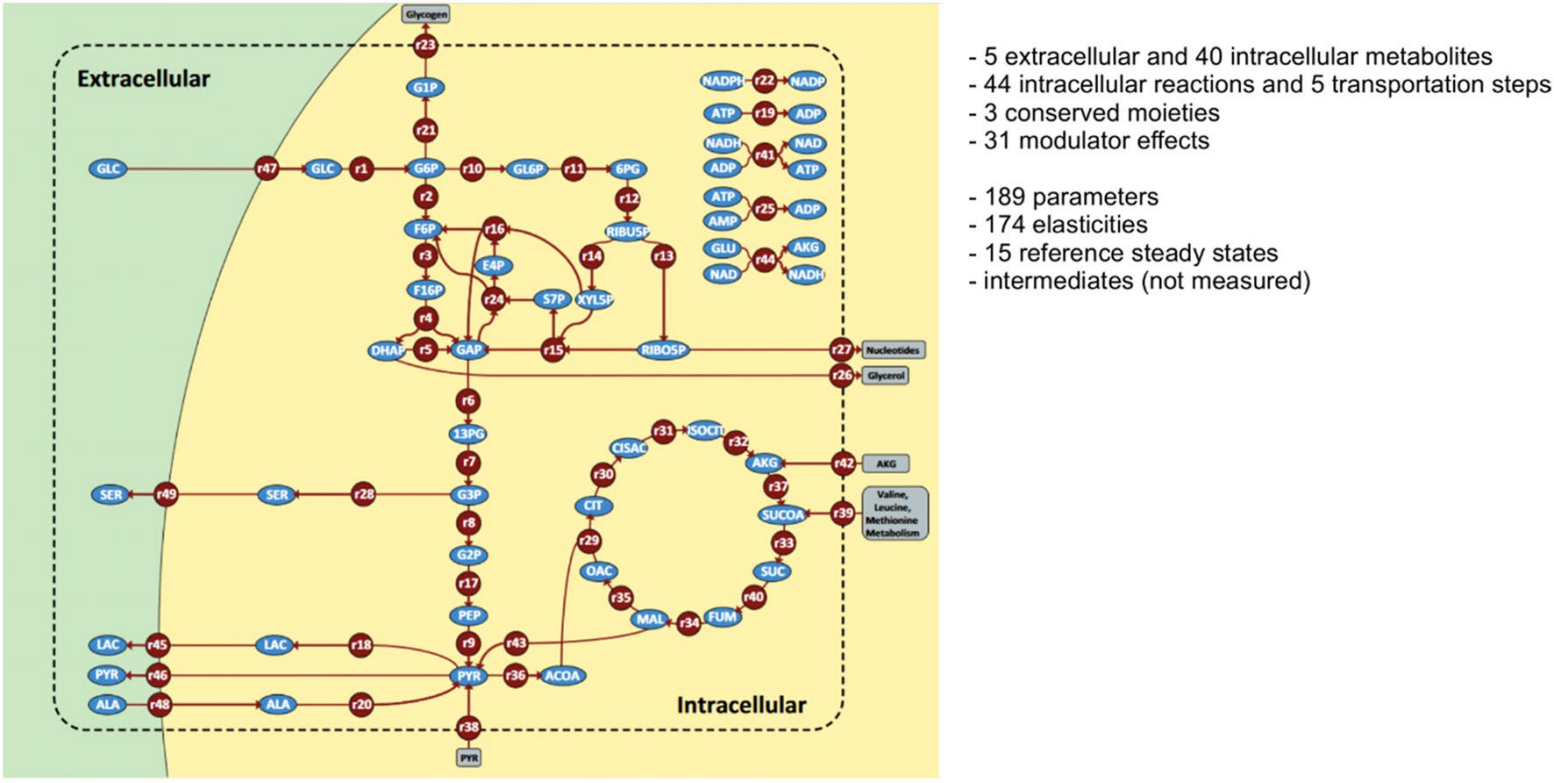
Metabolic network model (HepG2 liver cancer cells). The figure shows the model that is implemented in our multiscale framework within this study. Reprinted from^53^ under a CC BY license, with permission from BMC Systems Biology, original copyright 2010.

We now summarize the model of central metabolism in HEPG2 cells, which is included at the subcellular scale for each cell in our simulation domain. This model was proposed by Maier et al.^53^ (also see Fig. 2), and includes reactions representing glycolysis, the pentose-phosphate shunt, the citric acid cycle, and respiration. Briefly, the following generic mass balance equation describes the time-dependent behaviour of the metabolites:

4 $$\frac{d}{dt}\left(\frac{\mathbf{c}}{{{\varvec{c}}}^{0}}\right) ={\left({\mathbf{c}}^{0}\right)}^{-1}\mathbf{N} \mathbf{r},$$

where: $\mathbf{N}$ denotes the stoichiometric matrix; $\mathbf{r}$ is the vector of rate constants; ${\mathbf{c}}^{0}$ is a square diagonal matrix with steady-state concentrations of metabolites along its main diagonal; and ${\mathbf{c}/\mathbf{c}}^{0}$ is a vector of normalized metabolite concentrations. The following general equation represents a typical kinetic equation of the lin-log model:

5 $$\frac{r}{{J}_{0}}=\frac{{c}_{E}}{{c}_{E}^{0}}\left(1+\sum_{i}{\varepsilon }_{S,i}\mathrm{ln}\frac{{c}_{S,i}}{{c}_{S,i}^{0}}+\sum_{j}{\varepsilon }_{P,j}\mathrm{ln}\frac{{c}_{P,j}}{{c}_{P,j}^{0}}+\sum_{k}{\varepsilon }_{A,k}\mathrm{ln}\frac{{c}_{A,k}}{{c}_{A,k}^{0}}+\sum_{l}{\varepsilon }_{I,l}\mathrm{ln}\frac{{c}_{I,l}}{{c}_{I,l}^{0}}\right)$$

Here, subscripts denote participating species in the reaction, that is, substrates (*S*), products (*P*), activators (*A*) and inhibitors (*I*). Superscripts (0) in Eqs. (4) and (5) denote the reference state (for instance, steady state or initial condition). ${J}_{0}$ is the reference flux through this reaction, ${c}_{E}$ is the concentration of the enzyme catalysing the reaction, and the elasticity coefficients are defined as

6 $${\varepsilon }_{M}= \frac{{c}_{{M}_{0}}}{{r}_{0}}{\left(\frac{\partial {r}_{M}}{\partial {c}_{M}}\right)}_{0},$$

where $M\in \left\{\left(S,i\right),\left(P,j\right),\left(A,k\right),\left(I,l\right)\right\}$. The quantitative information about the steady-state metabolic flux analysis was derived with the aid of an instationary C-13 metabolic flux analysis^32,33^. The dynamic model was experimentally validated with quantitative measurements of 25 extracellular and intracellular intermediates during stimulus response experiments. For more details we refer to the original publications^32,33,53^.

### Coupling of intracellular and extracellular scales

The intracellular models are coupled to the extracellular environment through the concentration of the diffusible substances. Extracellular oxygen acts directly on the cell-cycle and the intracellular VEGF/p53 model. The feedback from the intracellular to the diffusible scale is implemented via secretion of VEGF (as the source term for the VEGF-PDE). Glucose is coupled to the metabolic network via a frozen steady-state and a one-way coupling (from extracellular to intracellular). Therefore, the local concentration values of glucose at the cell position are used to update the intracellular ODE model and to proceed in time. The coupling is also visualized in Fig. 1.

### Computational framework

The computational framework used to simulate the hybrid multiscale model of vascularized tumour growth is described in detail in^56,57^. In^57^ the model was extended from 2D to 3D and it was necessary to adapt the computational algorithm to the additional degree of freedom. Simulating large tumours is challenging due to the added computational demands created by the third dimension. Issues of memory allocation arise due an increase in the number of agents (cells) in model simulations. The inherent stochasticity of our hybrid model would require averaging over multiple realizations to extract robust conclusions. The latter aspects were neglected in the simulation results presented below, due to the large computational effort. The results presented are therefore representative results that show the underlying mechanisms of our model.

**Figure 3:**
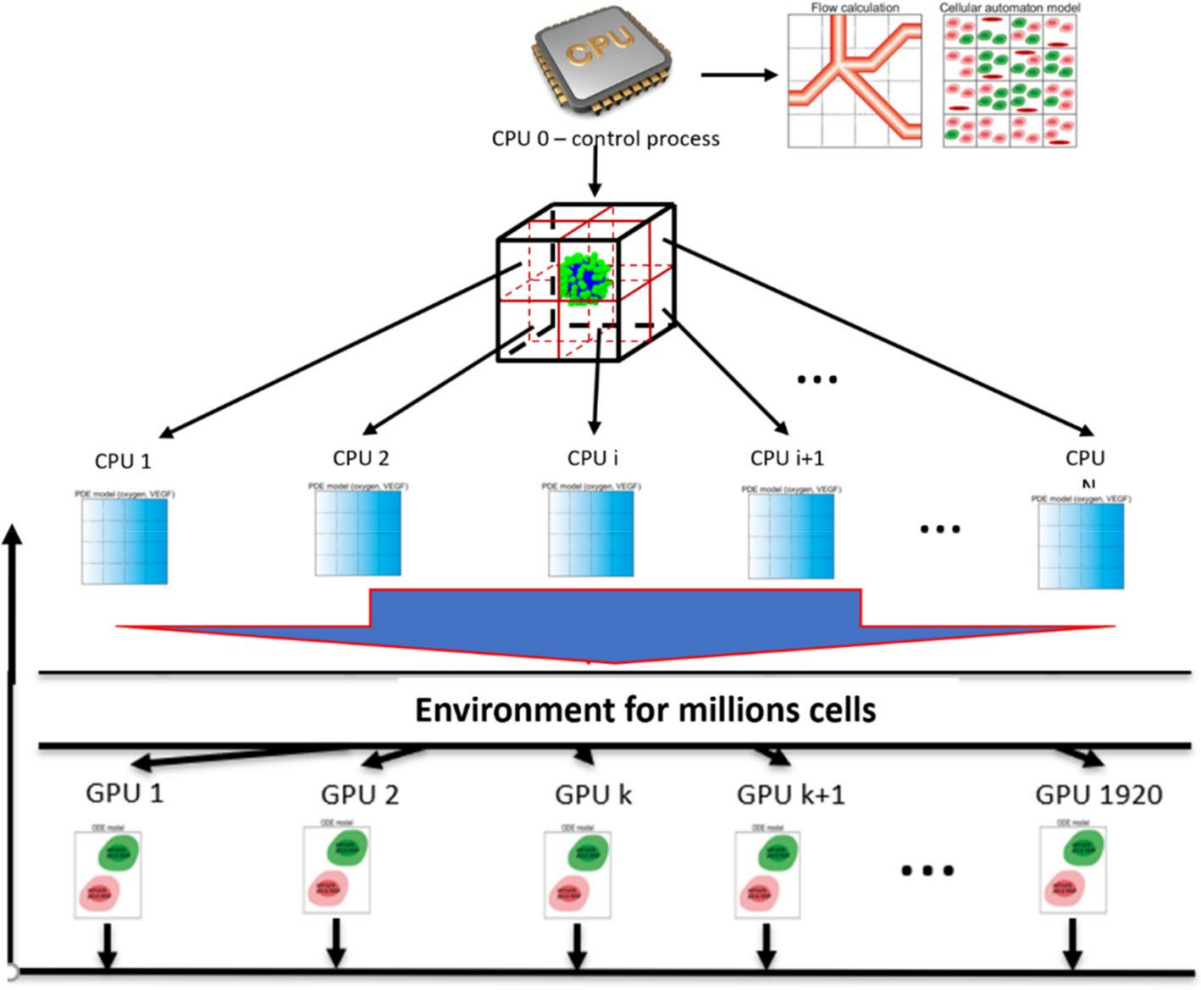
CPU-GPU systems for simulation. Strategy with parallelized CPU-GPU systems for simulation of the hybrid multiscale models for vascularized tumour growth. The extracellular concentrations (glucose/oxygen) are used to update the intracellular network and therefore the synchronization of both models takes place after the update of diffusible substances.

To master the remaining challenges regarding computer demand we follow the strategy of structural consistency between the multiscale structure of the model and the architecture of the computer hardware^66,67^, thereby utilizing the strengths of diverse computational hardware. A closer look at the structure of the model (Fig. 1) suggests the choice of a hybrid parallelized CPU (Central Processor Unit)—GPU (Graphical Processor Unit) system. After detailed investigations of the required computer demand of the individual modules shown in Fig. 1, we arrive at an optimal distribution of various tasks, as illustrated in Fig. 3.

The system of ODEs describing cell-cycle dynamics at the subcellular level was solved using the modified Bulirsch-Stoer method^68^. Since these ODEs decouple between agents and are only influenced by extracellular diffusible species such as oxygen (solved to quasi steady state), GPU-based methods are optimal. An adaptation of CUDA-FORTRAN was required to implement the modified Bulirsch-Stoer method on parallelized GPUs.

PDEs governing the diffusive transport and reactions governing extracellular oxygen, VEGF and glucose concentrations were discretized using the second order Alternating Direction Implicit method^69^, and solved on parallelized CPUs. The force-balance equations describing blood flow in the vascular network were solved on the CPU using the Liebmann successive displacement method^70^. The biased random walk of sprout tips resulting in angiogenesis was also implemented on a single CPU. Model simulations were visualized using the Coin3D implementation of the Open Inventor Application Programming Interface (API) and OpenGL^71^.

The dynamic model for the central metabolism in HEPG2 cells was numerically integrated using the LIMEX solver^72^. Since the time constants of the reactions in the metabolic network model (Eqs. (4) and (5)) are much shorter than cell division times, we assume that the simulations of the metabolic network can be based upon a frozen steady state of the vascularized tumour growth.

All simulation parameters, applied here, are given in Perfahl et al.^57^ and Meier et al.^33^.

## Results

### 3D vascular tumour growth

**Figure 4:**
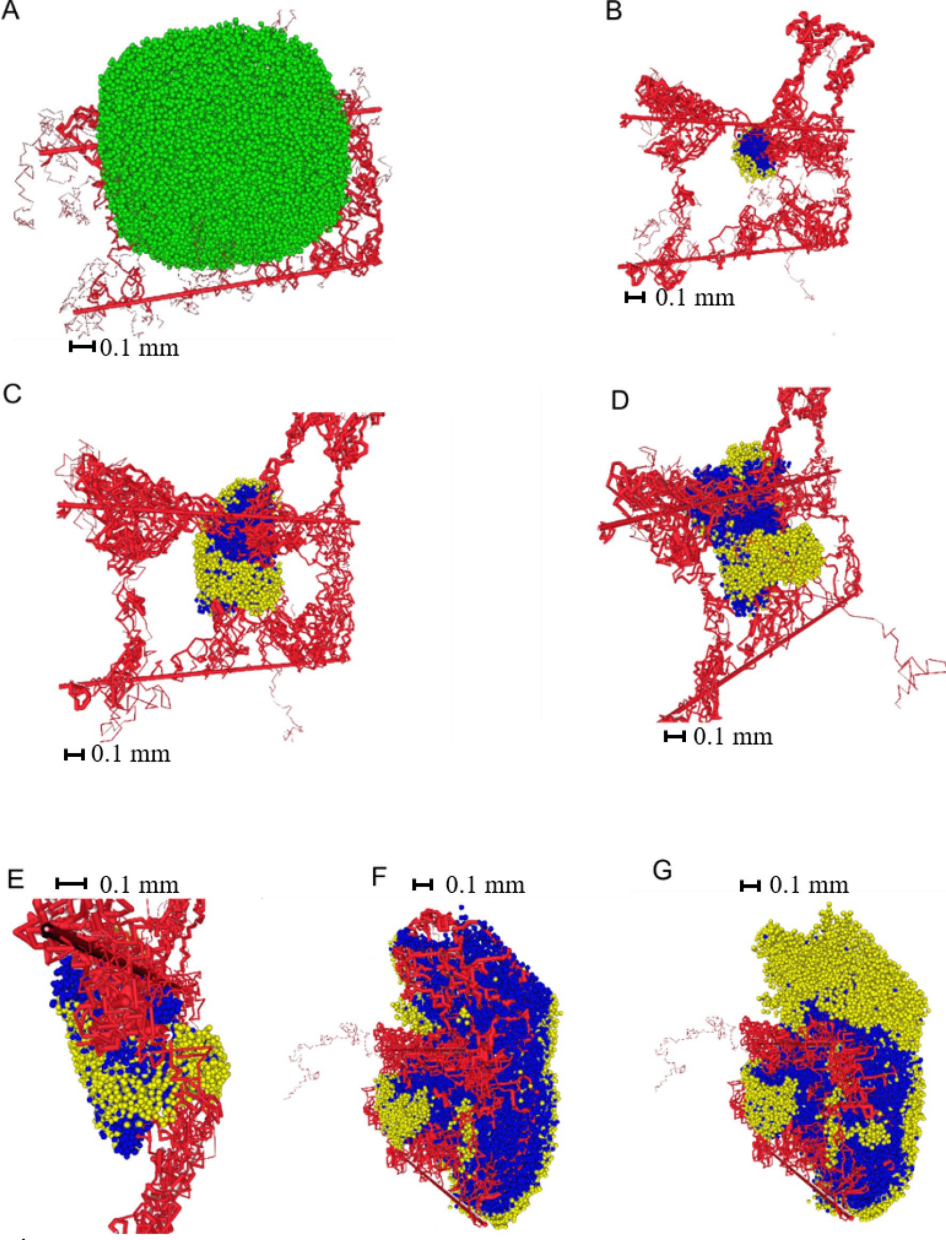
Three-dimensional growth of the tumour. (**A**) Healthy tissue embedded between 2 parent vessels results in efficient angiogenesis and a sufficient oxygen supply. (**B**) The initial tumour is implanted in the healthy tissue. For ease of visualization, only tumour cells are shown (blue: proliferating tumour cells, yellow: quiescent tumour cells) and healthy cells are hidden. (**C–E**) Further vascular tumour growth. (**F,G**) Different views of the tumour at final time point (**E**).

We first simulated the growth of a vascular tumour in three dimensions, to illustrate the effect of the new approach for capturing anastomosis. A typical model simulation is shown in Fig. 4. Simulations were performed on a 64/64/64 lattice with a spacing of 20 µm, which corresponds to a 1.28 mm × 1.28 mm × 1.28 mm cube of tissue. In these simulations, each lattice site can only be occupied by at most one cell (healthy or cancerous). Prior to tumour initiation, we assumed that the simulation domain was perfused by two parent vessels with countercurrent flow, that is, flows are in opposite directions. We then allowed healthy tissue and its associated vascular network—fed by the parent vessels—to grow into and finally occupy this domain (Fig. 4A). At time t = 0, a small tumour was implanted within the healthy tissue (Fig. 4B). As the tumour cells proliferated, they became hypoxic, resulting in VEGF secretion, a high degree of angiogenesis, and further tumour growth (Fig. 4C–E). We remark that our choice of the distance-dependent functional form of anastomosis probability results in a highly efficient process of *functional* vascular network formation in three dimensions.

### Integration of the dynamic model for central metabolism in individual cells with the vascular tumour growth model

**Figure 5:**
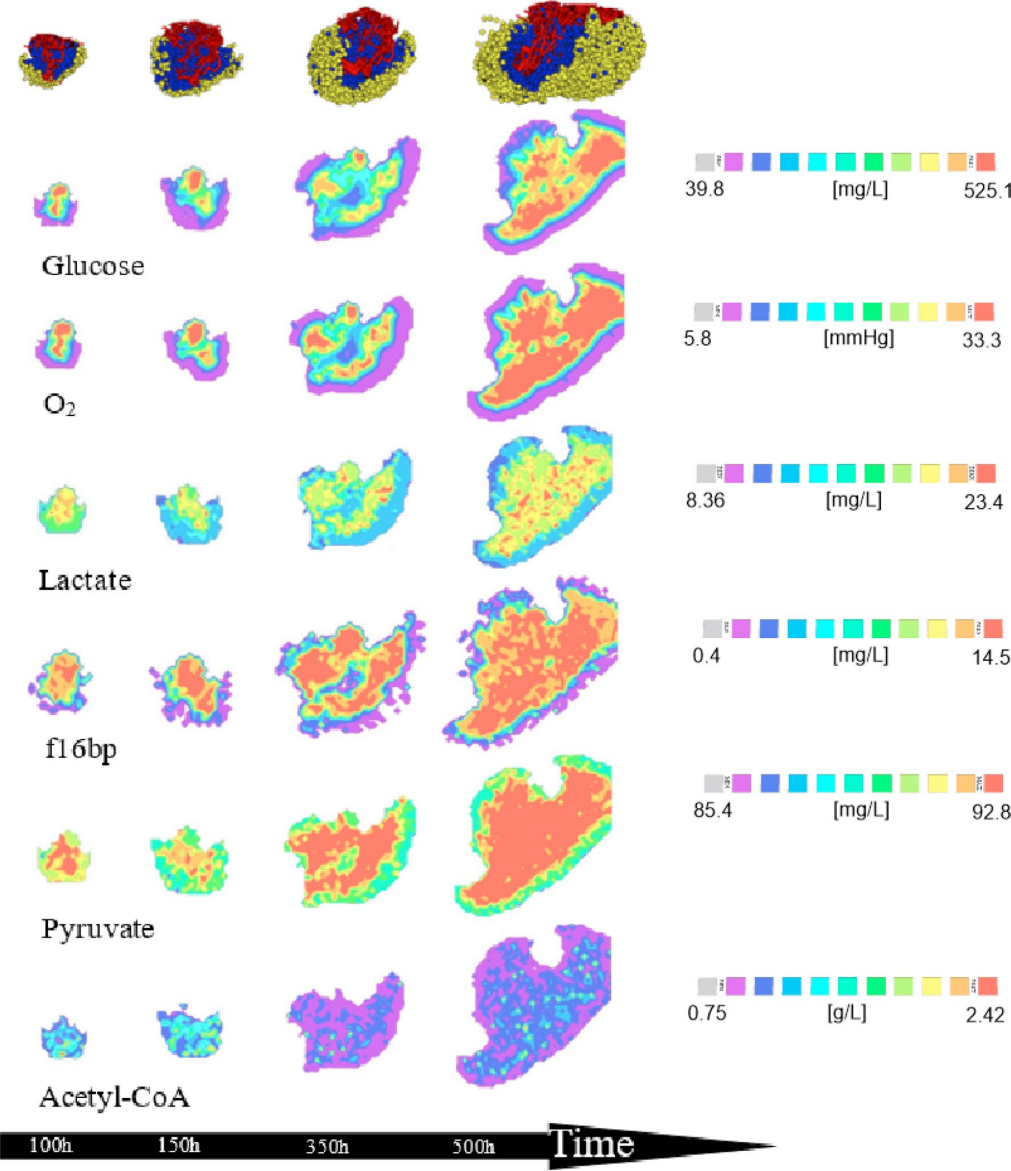
3D concentration fields. Equatorial cross-sections taken from 3D concentration fields of metabolite concentrations within the growing tumour (first row).

The central metabolism model was integrated into the 3D model of vascularized tumour growth as described in the methods section. Since reaction rates in the metabolic network model (Eqs. (4) and (5)) are much larger than cell division times, we assume that on the timescale of tumour growth, metabolite concentrations are at quasi steady state. This allowed us to capture the spatial distribution of key metabolites within the tumour, in three dimensions, and at each time point. The results of these simulations are shown in Fig. 5. The first row indicates vascular tumour growth showing proliferating (blue) and quiescent (yellow) tumour cells and intra-tumoural blood vessels (red). Equatorial cross-sections taken from the corresponding 3D concentration fields of metabolites (glucose, oxygen, lactate, fructose-1,6-bisphosphate, pyruvate, Acetyl-CoA) are shown in the remaining rows.

The spatio-temporal distributions of 2 nutrients, glucose and oxygen, mirror the increase in vessel density caused by tumour-induced angiogenesis. Briefly, hypoxia and hypoglycemia in poorly vascularized areas of the tumour induced VEGF secretion by tumour cells. The resulting angiogenesis largely restored nutrient supply, leaving only a smaller outer region that was nutrient deficient, as reflected by a thin layer of quiescent tumour cells. Visual validation of our model comes from the predicted concentration profiles of lactate within the tumour. Elevated lactate levels closely mirror high glucose levels (Fig. 5 rows 2 and 4), demonstrating the well-established Warburg effect, wherein cancer cells perform aerobic glycolysis—of which lactate is a by-product—even in the presence of oxygen^73^

In the initial stages of tumour growth, the concentrations of fructose-1,6-bisphosphate (f16bp) and pyruvate are predicted to be highly spatially heterogenous. In contrast, the citric acid cycle input, Acetyl-CoA, demonstrates less variability across the tumour. The relatively uniform Acetyl-CoA concentration is explained by the conversion of excess pyruvate (the precursor of Acetyl-CoA) into lactate, so that spatial gradients of lactate vary even though those of Acetyl-CoA do not. We remark that a relatively constant Acetyl-CoA concentration will lead to a steady rate of flux through the citric acid cycle, assuring ready availability of important precursors e.g. for the synthesis of fatty acids, in cancer cells^74^.

Visual validation for our model comes from comparing predictions of glucose concentration profiles with experimental data reported in^75^. The predicted heterogeneity in glucose concentration in our model simulations is in good qualitative agreement with PET scan measurements of ^18^F-FDG taken from a xenograft model of non-small cell lung cancer^75^. ^18^F-FDG uptake is directly related to glucose metabolism in lung cancer^76^.

**Figure 6:**
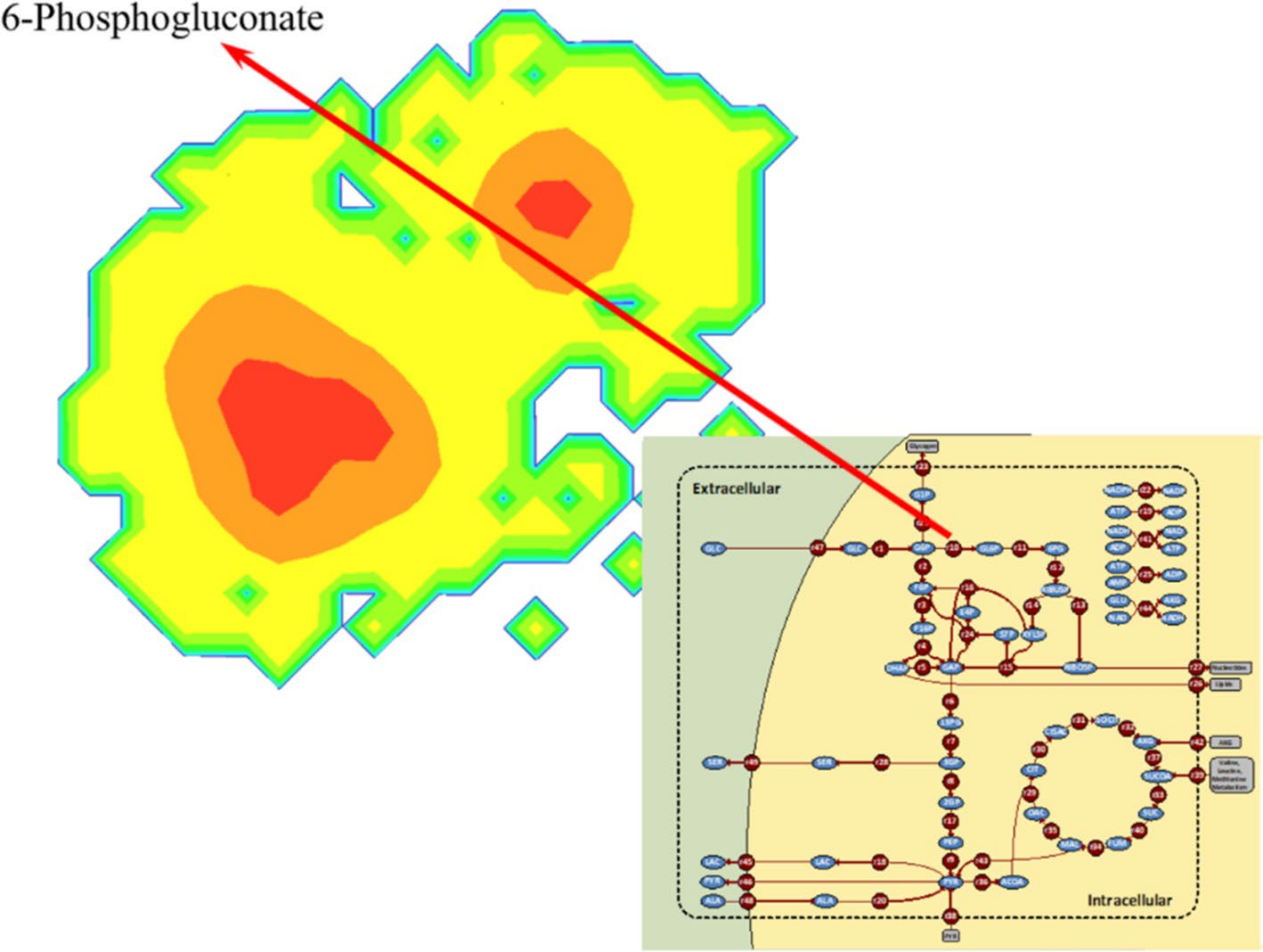
Spatial distribution of 6-phopshogluconate. The inhibition of glucose-6-phosphate dehydrogenase strongly influences cancer cell proliferation^77–79^ and may restore sensitivity of cancer cells to chemotherapy^80^. The figure shows schematically the structure of the spatial distribution of 6-phopshogluconate and the line points to its position in the metabolic network. Reprinted from^53^ under a CC BY license, with permission from BMC Systems Biology, original copyright 2010.

We also plot the spatial distribution of 6-phosphogluconate, an intermediary of the pentose-phosphate pathway, in Fig. 6. As expected, concentrations of metabolic intermediates associated with high levels of flux through the corresponding pathways are higher in rapidly dividing regions of the simulated tumour. The glucose flux through the pentose-phosphate pathway is much higher than would be needed to produce ribose 5-phosphate for DNA synthesis in support of cancer cell replication^32,33,53^. Our simulation results reflect this increased flux by the 6-phosphogluconate concentration, which provides biological support to our model because NADPH produced by this pathway is used by cancer cells to scavenge reactive oxidative species (ROS). Additionally, rapidly dividing cancer cells require NADPH/reducing potential for anabolic reactions such as lipid and cholesterol synthesis^74^.

**Figure 7:**
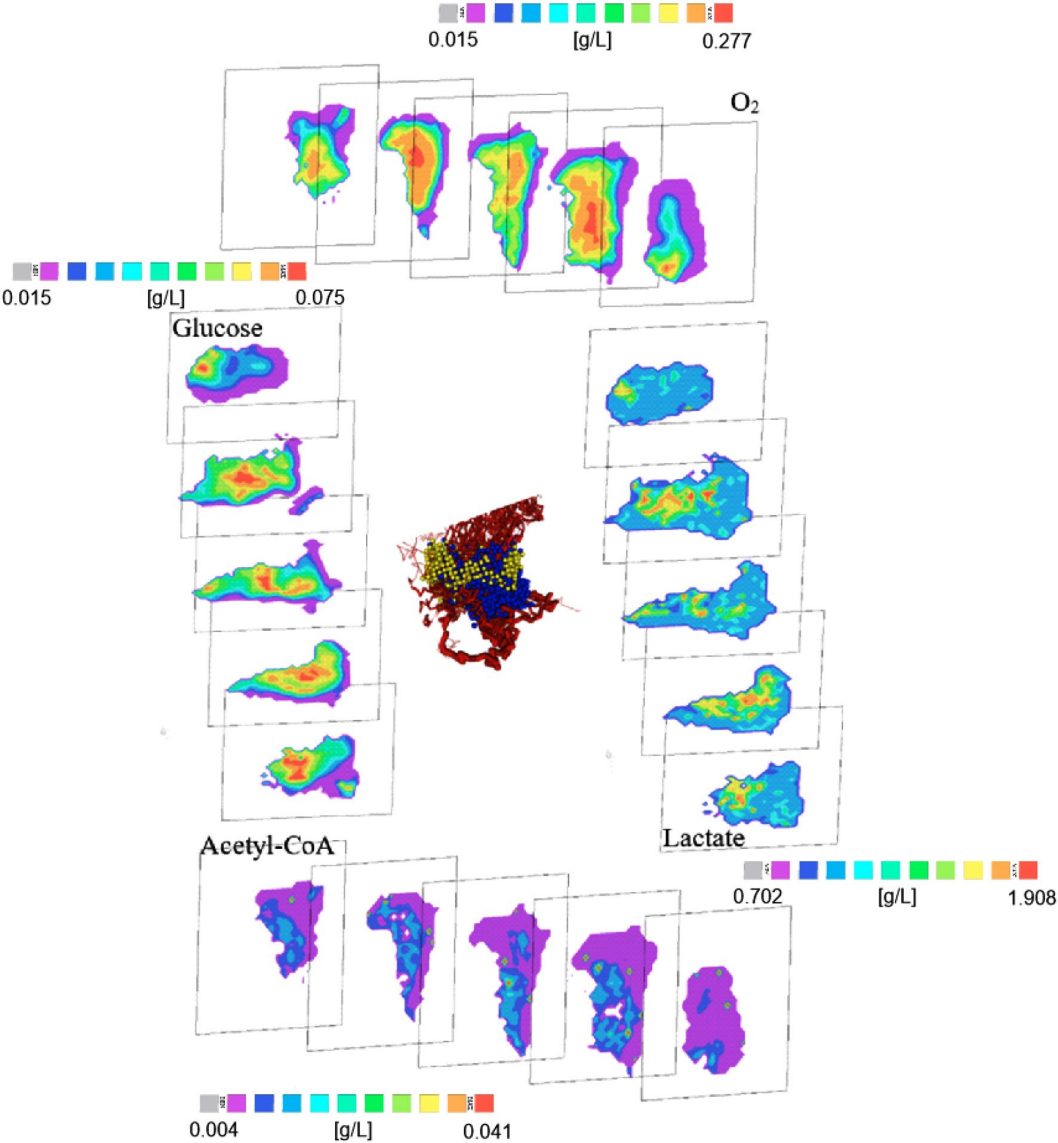
Examples of metabolite concentrations with increasing size of tumour. The pictures are slices with different offsets in the same time moment—corresponding to Fig. 5 time is approximately 350 h.

To illustrate the potential of our model in elucidating cancer metabolism, we plot three-dimensional distributions for selected metabolites in Fig. 7. These distributions illustrate for the first time the unexpected extent of spatial heterogeneity in cancer metabolism for different cell types in the local microenvironment.

## Conclusions

Variability in oxygen and glucose concentrations drive heterogeneity in the metabolic phenotype of cancer cells during tumour progression. This can significantly impact tumour response to anti-neoplastic agents, especially when drug response is dependent on the metabolic state of individual cancer cells^54^. Therefore, our over-arching goal is to bridge the conceptual gap between tumour-level metabolic processes, and the known metabolic processes occurring within individual cancer cells. To this end, we extended a computational framework here wherein a dynamic metabolic model was integrated with a hybrid 3D multiscale model of vascularized tumour growth.

We reported here the first three-dimensional model of intracellular metabolism dynamics as they respond to microenvironmental cues within a heterogeneous growing tumour. Model simulations predicted a high degree of spatial heterogeneity in key metabolites within the growing tumour. Such models, once validated, can be employed in the future to optimize targeted metabolism-based therapies.

For instance, the module for the dynamic model of metabolism can be used to carry out a model-based analysis (e.g. with the help of metabolic control analysis (MCA)) to identify suitable targets for drug development^53^. The need for experimentally verified dynamic models of cancer metabolism on which to test novel therapeutics has been highlighted in one of the first reviews focused on this topic^81^. In addition to identifying the main controlling steps for targeted drug development, our approach for linking the 3D vascularized tumour model with the dynamic metabolic model permits simulation and optimization of different therapeutic application of such drugs. The objective of these simulations could be the development of treatment strategies as a function of the progression of the tumour, repeating therapies and /or design of combination therapies in the case of treatments that combine more than one therapeutic agent. In spite of problems in clinical trials for treatment with metabolic inhibitors there are promising results in the literature for improving the application of mathematical tumour models for personalized tumour growth prediction. A recently published review^82^ focusing at the expert opinion on the topic of clinical application of these inhibitors reveals that although there is an increasing number of publications on the subject of metabolic inhibitors, the majority of these approaches are restricted to preclinical studies (particularly cell cultures) and only a few of them have been successfully transformed into clinical applications of cancer treatment. The important conclusion of the authors: “Toxicity of normal cells and high dosage required for the current inhibitors remain the showstopper”. As such, the necessary data from clinical trials are not available and we are unable to compensate for this deficit. Our concept for the application of the model for clinical application aims at simulations of alternative strategies to solve the above- mentioned problems of toxicological effects on healthy cells. A promising possibility for liver tumours would be the application of direct injection into the arterial inflow, as is already practiced with TACE therapy. We have previously used the tumour model for satisfactorily simulations of this therapy^62^. To improve the model-based predictions, we are currently working on expanding the modeling of the hexagonal blood supply of the liver module. This significant improvement in the imaging of vascularization is currently being incorporated in a further manuscript. Finally, it is planned to couple these model extensions with the dynamic model of the metabolism and to use them for the simulation of clinical therapies for the utilization of the inhibitors.

An important – and yet often overlooked – aspect in cancer models is the huge information gap between phenotype and genotype. That is, how gene expression and molecules influence the behaviour of a (cancer) cell^14^. This is a complex problem, not least since the mapping from genes to phenotype is not one-to-one but is probably many-to-one or even many-to-many. Sophisticated mathematical modeling could potentially help elucidate this “genotype-to-phenotype mapping”. Consequently, planned future work aims to connect the models developed here, with additional genetic sources of intra-tumour heterogeneity and extend the model to genetically and metabolically diverse tumour types. Most experiments and integrated computer models jointly tackling metabolism and genetics are carried out in microorganisms where parameter values are easier to acquire^83–88^. However, the results reported here can motivate targeted experiments on metabolism and cancer cell genetics, which can in turn be used to refine the model.

Complementing these advances at the cellular level, tissue-level experiments are needed to validate the vascular component of these hybrid models. Yankeelov et al.^89,90^ have noted the challenges in using clinical imaging data for this purpose. Nonetheless, significant advances in experimental methods to image metabolic processes in three-dimensions^91–93^ render validation of the simulation results of the comprehensive model feasible.

## Data Availability

The datasets used and/or analysed during the current study available from the corresponding author on reasonable request.

## Code availability

All algorithm used to generate the results that are reported in the paper and central to its main claims are fully published under^53,56,57^.
